# Supplementary material for: Effects of various living-low and training-high modes with distinct training prescriptions on sea-level performance: A network meta-analysis
Source: PLoS One. 2024 Apr 18;19(4):e0297007. doi: 10.1371/journal.pone.0297007 (PMC11025749; doi:10.1371/journal.pone.0297007)
Supplement: S1 Table — (DOCX) [file pone.0297007.s002.docx]

**Supporting information file 3: Search Strategy**

## Database: web of science<inception to June 30, 2023>

| PARTICIPANTS | (TS=(athletes ) |
| --- | --- |
| INTERVENTION | ((((((((((TS=(hypoxia*)) OR TS=(hypoxic training)) OR TS=(altitude training)) OR TS=(hypoxia training)) OR TS=(intermittent hypoxic exposure)) OR TS=(intermittent hypoxic training)) OR TS=(interval hypoxic training)) OR TS=(interval training in hypoixa)) OR TS=(repeated sprint training in hypoxia)) OR TS=(interval sprint training in hypoxia)) OR TS=(continuous hypoxic training) |
| OUTCOMES | ((((((((TS=(aerobic performance)) OR TS=(anaerobic performance)) OR TS=(endurance)) OR TS=(incremental treadmill test)) OR TS=(3 min All-Out Test)) OR TS=(YYIR test)) OR TS=(Field test)) OR TS=(Wingate Test)) OR TS=(Maximal anaerobic test) |
| STUDY DESING | ((((((TS=(randomised )) OR TS=(randomized )) OR TS=(randomisation )) OR TS=(randomisation )) OR TS=(placebo)) OR (TS=(random)) AND (TS=(allocat)) OR TS=(assign)) OR (TS=(blind)) AND (((TS=(single )) OR TS=(double )) OR TS=(treble )) OR TS=(triple) |
| Note: Keyword groups will be linked by AND | |
