# Supplementary material for: Effects of various living-low and training-high modes with distinct training prescriptions on sea-level performance: A network meta-analysis
Source: PLoS One. 2024 Apr 18;19(4):e0297007. doi: 10.1371/journal.pone.0297007 (PMC11025749; doi:10.1371/journal.pone.0297007)
Supplement: S2 Table — (DOCX) [file pone.0297007.s003.docx]

**Supporting information table 7: Physiotherapy Evidence Database (PEDro) scores of the included studies**

| study | Eligibility criteria | Random allocation | Concealed allocation | Baseline comparability | Blind subjects | Blind therapists | Blind assessor | Adequate follow-up dropout  \15 % | Intention-to-treat analysis | Between-  group comparisons | Point estimates and variability | Score |
| --- | --- | --- | --- | --- | --- | --- | --- | --- | --- | --- | --- | --- |
| S.R. Goods et al. 2015 | + | + | - | + | + | - | - | + | + | + | + | 7 |
| Giovanna et al. 2022 | + | + | + | + | - | - | - | + | + | + | + | 7 |
| Kasai et al. 2015 | - | + | - | + | + | - | - | + | + | + | + | 7 |
| Wadee et al. 2022 | + | + | - | + | + | - | - | + | + | + | + | 7 |
| Monteroet al. 2016 | + | + | - | - | + | + | + | + | + | + | + | 8 |
| Faiss et al. 2013 | + | + | - | + | + | + | - | + | + | + | + | 8 |
| M Galvin et al. 2013 | + | + | - | - | + | - | - | - | + | + | + | 5 |
| Gatterer et al. 2014 | + | + | - | - | + | - | - | - | + | + | + | 5 |
| Faiss et al. 2015 | + | + | - | + | + | + | - | + | + | + | + | 8 |
| Brocherie et al. 2015 | + | + | - | + | + | + | - | + | + | + | + | 8 |
| Brocherie et al. 2015 | + | + | - | + | + | + | - | + | - | + | + | 7 |
| Brechbuhl et al. 2020 | + | + | - | + | + | - | - | + | + | + | + | 7 |
| Kasaiet al. 2017 | + | + | - | + | + | + | - | + | + | + | + | 8 |
| Wang et al. 2018 | + | + | - | + | + | + | - | + | - | + | + | 7 |
| Shi et al. 2023 | + | + | - | + | + | - | - | + | + | + | + | 7 |
| Gatterer et al. 2018 | - | + | - | - | - | - | - | - | + | + | + | 4 |
| Warnier et al. 2020 | + | + | - | - | + | - | - | + | + | - | + | 5 |
| Karabiyik et al. 2021 | + | + | + | + | + | - | - | - | + | + | + | 7 |
| Truijens et al. 2002 | - | + | - | - | + | + | - | + | - | + | + | 6 |
| Arezzolo et al. 2020 | - | + | - | + | + | - | - | + | + | + | + | 7 |
| Ambrozy et al. 2020 | + | + | - | + | - | - | - | + | + | + | + | 6 |
| Morton et al. 2005 | - | + | - | - | - | - | - | + | + | + | + | 5 |
| Roels et al. 2005 | + | + | - | - | - | - | - | + | + | + | + | 5 |
| Holliss et al. 2014 | - | + | - | + | + | - | - | - | + | - | + | 5 |
| Hendriksen et al. 2003 | + | - | - | - | - | - | - | + | + | + | + | 4 |
| Ponsot et al. 2005 | - | + | - | + | + | - | - | + | + | + | + | 7 |
| Zoll et al. 2005 | + | + | - | + | - | - | - | + | + | + | + | 6 |
| Czuba et al. 2017 | - | + | - | - | - | - | - | + | + | + | + | 5 |
| Park et al. 2022 | + | + | - | + | - | - | - | + | + | + | + | 6 |
| Morris et al. 2020 | - | + | - | - | + | - | - | + | + | + | - | 5 |
| Jung et al. 2020 | - | - | - | + | - | - | - | + | + | + | + | 5 |
| Millet et al. 2013 | - | + | - | + | - | - | - | + | + | + | + | 6 |
| Czuba et al. 2011 | - | + | - | - | - | - | - | + | + | + | + | 5 |
| Czuba et al. 2018 | - | + | - | + | - | - | - | + | + | + | + | 6 |
| Czuba et al. 2019 | - | + | - | - | + | - | - | + | + | + | + | 6 |
| Dufour et al. 2005 | + | + | - | + | - | - | - | + | + | + | + | 6 |
| Sanchez et al. 2018 | - | + | - | - | + | + | - | + | + | - | + | 6 |
| Ramos-Campo et al. 2015 | - | + | - | - | - | - | - | + | + | + | + | 5 |
| Roels et al. 2007 | - | + | - | - | - | - | - | + | + | + | + | 5 |
| Millet et al. 2014 | - | + | - | + | - | - | - | + | + | + | + | 6 |
| Kim et al. 2021 | - | + | - | + | - | - | - | + | + | + | + | 6 |
| Robach et al. 2014 | - | + | - | - | + | + | - | + | + | + | + | 7 |
| Hamlin et al. 2010 | + | + | - | + | + | - | - | + | + | + | + | 7 |
| Park et al. 2018 | + | - | - | - | - | - | - | + | + | + | + | 4 |
| Julian et al. 2003 | + | + | - | + | + | + | - | + | + | + | + | 8 |
| Hinckson et al. 2006 | - | + | - | + | + | + | - | + | + | + | + | 8 |
| Katayama et al. 2004 | + | + | - | - | - | - | - | + | + | + | + | 5 |
| Rodríguez et al. 2014 | + | + | - | + | + | + | - | + | + | + | + | 8 |
| Tadibi et al. 2007 | - | + | - | - | + | + | - | + | + | + | + | 7 |
| Miller et al. 2014 | - | + | - | - | + | + | - | + | + | + | + | 7 |
| Gough et al. 2019 | + | + | - | + | + | + | - | + | + | + | + | 8 |
| Burtsche et al. 2010 | - | + | - | + | + | + | - | + | + | + | + | 8 |
| Katayama et al. 2003 | - | + | - | + | + | + | - | + | + | + | + | 8 |
| Bonetti et al. 2006 | - | + | - | - | + | - | - | + | + | + | + | 6 |
| Lázaro et al. 2002 | - | + | - | + | + | - | - | + | + | + | + | 7 |
| Hamlin et al. 2002 | - | + | - | - | + | - | - | + | + | + | + | 6 |

Table 3. Physiotherapy Evidence Database (PEDro) scores of the included studie
