# Supplementary material for: Effects of various living-low and training-high modes with distinct training prescriptions on sea-level performance: A network meta-analysis
Source: PLoS One. 2024 Apr 18;19(4):e0297007. doi: 10.1371/journal.pone.0297007 (PMC11025749; doi:10.1371/journal.pone.0297007)
Supplement: S3 Table — (DOCX) [file pone.0297007.s004.docx]

**Supporting information table 8: the included studies.**

| Study | hypoxic  protocol |
| --- | --- |
|  |  |
| S.R. Goods et al. 2015[31] | RSH |
| Giovanna et al. 2022[32] | RSH |
| Kasai et al. 2015[33] | RSH |
| Wadee et al. 2022[34] | RSH |
| Montero et al. 2016[35] | RSH |
| Faiss et al. 2013[36] | RSH |
| M Galvin et al. 2013[37] | RSH |
| Gatterer et al. 2014[38] | RSH |
| Faiss et al. 2015[39] | RSH |
| Brocherie et al. 2015[40] | RSH |
| Brocherie et al. 2015[41] | RSH |
| Brechbuhl et al. 2020[42] | RSH |
| Kasaiet al. 2017[43] | RSH |
| Wang et al. 2018[44] | RSH |
| Shi et al. 2023[45] | RSH |
| Gatterer et al. 2018[46] | RSH ISH |
| Warnier et al. 2020[47] | ISH |
| Karabiyik et al. 2021[48] | ISH |
| Ramos-Campo et al. 2015[49] | CHT+IHT |
| Truijens et al. 2002[50] | s-IHT |
| Ponsot et al. 2005[51] | l-IHT |
| Julian et al. 2003[52] | IHE |
| Roels et al. 2005[53] | CHT s-IHT |
| Roels et al. 2007[54] | CHT+IHT |
| Zoll et al. 2005[55] | l-IHT |
| Arezzolo et al. 2020[56] | s-IHT |
| Czuba et al. 2017[57] | l-IHT |
| Park et al. 2022[58] | l-IHT |
| Hinckson et al. 2006[59] | IHE |
| Morris et al. 2020[60] | l-IHT |
| Jung et al. 2020[61] | l-IHT |
| Millet et al. 2013[62] | l-IHT |
| Millet et al. 2014[63] | CHT+IHT |
| Czuba et al. 2011[64] | l-IHT |
| Holliss et al. 2014[65] | CHT |
| Czuba et al. 2018[66] | l-IHT |
| Ambrozy et al. 2020[67] | s-IHT |
| Morton et al. 2005[68] | s-IHT |
| Czuba et al. 2019[69] | l-IHT |
| Kim et al. 2021[70] | CHT+IHIT |
| Dufour et al. 2005[71] | l-IHT |
| Sanchez et al. 2018[72] | l-IHT |
| Robach et al. 2014[73] | CHT+IHT |
| Hamlin et al. 2010[74] | CHT+IHT |
| Katayama et al. 2004[75] | IHE |
| Park et al. 2018[76] | CHT+IHT |
| Rodríguez et al. 2014[77] | IHE |
| Tadibi et al. 2007[78] | IHE |
| Miller et al. 2014[79] | IHE |
| Gough et al. 2019[80] | IHE |
| Burtsche et al. 2010[81] | IHE |
| Hendriksen et al. 2003[82] | CHT |
| Katayama et al. 2003[75] | IHE |
| Bonetti et al. 2006[83] | IHE |
| Lázaro et al. 2002[84] | IHE |
| Hamlin et al. 2002[85] | IHE |

31. Goods PS, Dawson B, Landers GJ, Gore CJ, Peeling P. No Additional Benefit of Repeat-Sprint Training in Hypoxia than in Normoxia on Sea-Level Repeat-Sprint Ability. J Sports Sci Med. 2015;14(3):681-8. Epub 2015/09/04. PubMed PMID: 26336357; PubMed Central PMCID: PMCPMC4541135.

32. Giovanna M, Solsona R, Sanchez AMJ, Borrani F. Effects of short-term repeated sprint training in hypoxia or with blood flow restriction on response to exercise. J Physiol Anthropol. 2022;41(1):32. Epub 2022/09/04. doi: 10.1186/s40101-022-00304-1. PubMed PMID: 36057591; PubMed Central PMCID: PMCPMC9440585.

33. Kasai N, Mizuno S, Ishimoto S, Sakamoto E, Maruta M, Goto K. Effect of training in hypoxia on repeated sprint performance in female athletes. Springerplus. 2015;4:310. Epub 2015/07/15. doi: 10.1186/s40064-015-1041-4. PubMed PMID: 26155449; PubMed Central PMCID: PMCPMC4488237.

34. Pramkratok W, Songsupap T, Yimlamai T. Repeated sprint training under hypoxia improves aerobic performance and repeated sprint ability by enhancing muscle deoxygenation and markers of angiogenesis in rugby sevens. Eur J Appl Physiol. 2022;122(3):611-22. Epub 2022/01/04. doi: 10.1007/s00421-021-04861-8. PubMed PMID: 34977961.

35. Montero D, Lundby C. No Improved Performance With Repeated-Sprint Training in Hypoxia Versus Normoxia: A Double-Blind and Crossover Study. Int J Sports Physiol Perform. 2017;12(2):161-7. Epub 2016/05/04. doi: 10.1123/ijspp.2015-0691. PubMed PMID: 27140941.

36. Faiss R, Léger B, Vesin JM, Fournier PE, Eggel Y, Dériaz O, et al. Significant molecular and systemic adaptations after repeated sprint training in hypoxia. PLoS One. 2013;8(2):e56522. Epub 2013/02/26. doi: 10.1371/journal.pone.0056522. PubMed PMID: 23437154; PubMed Central PMCID: PMCPMC3577885.

37. Galvin HM, Cooke K, Sumners DP, Mileva KN, Bowtell JL. Repeated sprint training in normobaric hypoxia. Br J Sports Med. 2013;47 Suppl 1(Suppl 1):i74-9. Epub 2013/12/07. doi: 10.1136/bjsports-2013-092826. PubMed PMID: 24282212; PubMed Central PMCID: PMCPMC3903144.

38. Gatterer H, Philippe M, Menz V, Mosbach F, Faulhaber M, Burtscher M. Shuttle-run sprint training in hypoxia for youth elite soccer players: a pilot study. J Sports Sci Med. 2014;13(4):731-5. Epub 2014/12/02. PubMed PMID: 25435763; PubMed Central PMCID: PMCPMC4234940.

39. Faiss R, Willis S, Born DP, Sperlich B, Vesin JM, Holmberg HC, et al. Repeated double-poling sprint training in hypoxia by competitive cross-country skiers. Med Sci Sports Exerc. 2015;47(4):809-17. Epub 2014/08/02. doi: 10.1249/mss.0000000000000464. PubMed PMID: 25083727.

40. Brocherie F, Millet GP, Hauser A, Steiner T, Rysman J, Wehrlin JP, et al. "Live High-Train Low and High" Hypoxic Training Improves Team-Sport Performance. Med Sci Sports Exerc. 2015;47(10):2140-9. Epub 2015/02/11. doi: 10.1249/mss.0000000000000630. PubMed PMID: 25668402.

41. Brocherie F, Girard O, Faiss R, Millet GP. High-intensity intermittent training in hypoxia: a double-blinded, placebo-controlled field study in youth football players. J Strength Cond Res. 2015;29(1):226-37. Epub 2014/07/01. doi: 10.1519/jsc.0000000000000590. PubMed PMID: 24978836.

42. Brechbuhl C, Brocherie F, Willis SJ, Blokker T, Montalvan B, Girard O, et al. On the Use of the Repeated-Sprint Training in Hypoxia in Tennis. Front Physiol. 2020;11:588821. Epub 2021/01/12. doi: 10.3389/fphys.2020.588821. PubMed PMID: 33424620; PubMed Central PMCID: PMCPMC7793694.

43. Kasai N, Kojima C, Sumi D, Takahashi H, Goto K, Suzuki Y. Impact of 5 Days of Sprint Training in Hypoxia on Performance and Muscle Energy Substances. Int J Sports Med. 2017;38(13):983-91. Epub 2017/10/02. doi: 10.1055/s-0043-117413. PubMed PMID: 28965346.

44. Wang R, Fukuda DH, Hoffman JR, La Monica MB, Starling TM, Stout JR, et al. Distinct Effects of Repeated-Sprint Training in Normobaric Hypoxia and β-Alanine Supplementation. J Am Coll Nutr. 2019;38(2):149-61. Epub 2018/10/03. doi: 10.1080/07315724.2018.1475269. PubMed PMID: 30277420.

45. Shi Q, Tong TK, Nie J, Tao D, Zhang H, Tan X, et al. Repeated-sprint training in hypoxia boosts up team-sport-specific repeated-sprint ability: 2-week vs 5-week training regimen. Eur J Appl Physiol. 2023. Epub 2023/06/19. doi: 10.1007/s00421-023-05252-x. PubMed PMID: 37335354.

46. Gatterer H, Menz V, Salazar-Martinez E, Sumbalova Z, Garcia-Souza LF, Velika B, et al. Exercise Performance, Muscle Oxygen Extraction and Blood Cell Mitochondrial Respiration after Repeated-Sprint and Sprint Interval Training in Hypoxia: A Pilot Study. J Sports Sci Med. 2018;17(3):339-47. Epub 2018/08/18. PubMed PMID: 30116106; PubMed Central PMCID: PMCPMC6090395.

47. Warnier G, Benoit N, Naslain D, Lambrecht S, Francaux M, Deldicque L. Effects of Sprint Interval Training at Different Altitudes on Cycling Performance at Sea-Level. Sports (Basel). 2020;8(11). Epub 2020/11/22. doi: 10.3390/sports8110148. PubMed PMID: 33217937; PubMed Central PMCID: PMCPMC7698804.

48. Karabiyik H, Eser MC, Guler O, Yasli BC, Ertetik G, Sisman A, et al. The Effects of 15 or 30 s SIT in Normobaric Hypoxia on Aerobic, Anaerobic Performance and Critical Power. Int J Environ Res Public Health. 2021;18(8). Epub 2021/05/01. doi: 10.3390/ijerph18083976. PubMed PMID: 33918866; PubMed Central PMCID: PMCPMC8069352.

49. Ramos-Campo DJ, Martínez-Sánchez F, Esteban-García P, Rubio-Arias JA, Clemente-Suarez VJ, Jiménez-Díaz JF. The effects of intermittent hypoxia training on hematological and aerobic performance in triathletes. Acta Physiol Hung. 2015;102(4):409-18. Epub 2015/12/23. doi: 10.1556/036.102.2015.4.8. PubMed PMID: 26690033.

50. Truijens MJ, Toussaint HM, Dow J, Levine BD. Effect of high-intensity hypoxic training on sea-level swimming performances. J Appl Physiol (1985). 2003;94(2):733-43. Epub 2002/10/23. doi: 10.1152/japplphysiol.00079.2002. PubMed PMID: 12391107.

51. Ponsot E, Dufour SP, Zoll J, Doutrelau S, N'Guessan B, Geny B, et al. Exercise training in normobaric hypoxia in endurance runners. II. Improvement of mitochondrial properties in skeletal muscle. J Appl Physiol (1985). 2006;100(4):1249-57. Epub 2005/12/13. doi: 10.1152/japplphysiol.00361.2005. PubMed PMID: 16339351.

52. Julian CG, Gore CJ, Wilber RL, Daniels JT, Fredericson M, Stray-Gundersen J, et al. Intermittent normobaric hypoxia does not alter performance or erythropoietic markers in highly trained distance runners. J Appl Physiol (1985). 2004;96(5):1800-7. Epub 2003/12/16. doi: 10.1152/japplphysiol.00969.2003. PubMed PMID: 14672967.

53. Roels B, Millet GP, Marcoux CJ, Coste O, Bentley DJ, Candau RB. Effects of hypoxic interval training on cycling performance. Med Sci Sports Exerc. 2005;37(1):138-46. Epub 2005/01/06. doi: 10.1249/01.mss.0000150077.30672.88. PubMed PMID: 15632680.

54. Roels B, Bentley DJ, Coste O, Mercier J, Millet GP. Effects of intermittent hypoxic training on cycling performance in well-trained athletes. Eur J Appl Physiol. 2007;101(3):359-68. Epub 2007/07/20. doi: 10.1007/s00421-007-0506-8. PubMed PMID: 17636319.

55. Zoll J, Ponsot E, Dufour S, Doutreleau S, Ventura-Clapier R, Vogt M, et al. Exercise training in normobaric hypoxia in endurance runners. III. Muscular adjustments of selected gene transcripts. J Appl Physiol (1985). 2006;100(4):1258-66. Epub 2006/03/17. doi: 10.1152/japplphysiol.00359.2005. PubMed PMID: 16540710.

56. Arezzolo D, Coffey VG, Byrne NM, Doering TM. Effects of Eight Interval Training Sessions in Hypoxia on Anaerobic, Aerobic, and High Intensity Work Capacity in Endurance Cyclists. High Alt Med Biol. 2020;21(4):370-7. Epub 2020/08/25. doi: 10.1089/ham.2020.0066. PubMed PMID: 32830992.

57. Czuba M, Wilk R, Karpiński J, Chalimoniuk M, Zajac A, Langfort J. Intermittent hypoxic training improves anaerobic performance in competitive swimmers when implemented into a direct competition mesocycle. PLoS One. 2017;12(8):e0180380. Epub 2017/08/02. doi: 10.1371/journal.pone.0180380. PubMed PMID: 28763443; PubMed Central PMCID: PMCPMC5538675.

58. Park HY, Jung WS, Kim SW, Kim J, Lim K. Effects of Interval Training Under Hypoxia on Hematological Parameters, Hemodynamic Function, and Endurance Exercise Performance in Amateur Female Runners in Korea. Front Physiol. 2022;13:919008. Epub 2022/06/07. doi: 10.3389/fphys.2022.919008. PubMed PMID: 35665230; PubMed Central PMCID: PMCPMC9158122.

59. Hinckson EA, Hopkins WG, Downey BM, Smith TB. The effect of intermittent hypoxic training via a hypoxic inhaler on physiological and performance measures in rowers: a pilot study. J Sci Med Sport. 2006;9(1-2):177-80. Epub 2006/04/01. doi: 10.1016/j.jsams.2006.01.001. PubMed PMID: 16574484.

60. Morris DM, Kearney JT, Burke ER. The effects of breathing supplemental oxygen during altitude training on cycling performance. J Sci Med Sport. 2000;3(2):165-75. Epub 2000/12/05. doi: 10.1016/s1440-2440(00)80078-x. PubMed PMID: 11104308.

61. Jung WS, Kim SW, Park HY. Interval Hypoxic Training Enhances Athletic Performance and Does Not Adversely Affect Immune Function in Middle- and Long-Distance Runners. Int J Environ Res Public Health. 2020;17(6). Epub 2020/03/20. doi: 10.3390/ijerph17061934. PubMed PMID: 32188027; PubMed Central PMCID: PMCPMC7143158.

62. Czuba M, Zając A, Maszczyk A, Roczniok R, Poprzęcki S, Garbaciak W, et al. The effects of high intensity interval training in normobaric hypoxia on aerobic capacity in basketball players. J Hum Kinet. 2013;39:103-14. Epub 2014/02/11. doi: 10.2478/hukin-2013-0073. PubMed PMID: 24511346; PubMed Central PMCID: PMCPMC3916912.

63. Millet G, Bentley DJ, Roels B, Mc Naughton LR, Mercier J, Cameron-Smith D. Effects of intermittent training on anaerobic performance and MCT transporters in athletes. PLoS One. 2014;9(5):e95092. Epub 2014/05/07. doi: 10.1371/journal.pone.0095092. PubMed PMID: 24797797; PubMed Central PMCID: PMCPMC4010422 One colleague is employed by a commercial company “ORION, Clinical Services Ltd.” However, this does not alter the authors' adherence to PLOS ONE policies on sharing data and materials.

64. Czuba M, Waskiewicz Z, Zajac A, Poprzecki S, Cholewa J, Roczniok R. The effects of intermittent hypoxic training on aerobic capacity and endurance performance in cyclists. J Sports Sci Med. 2011;10(1):175-83. Epub 2011/01/01. PubMed PMID: 24149312; PubMed Central PMCID: PMCPMC3737917.

65. Holliss BA, Burden RJ, Jones AM, Pedlar CR. Eight weeks of intermittent hypoxic training improves submaximal physiological variables in highly trained runners. J Strength Cond Res. 2014;28(8):2195-203. Epub 2014/02/12. doi: 10.1519/jsc.0000000000000406. PubMed PMID: 24513622.

66. Czuba M, Fidos-Czuba O, Płoszczyca K, Zając A, Langfort J. Comparison of the effect of intermittent hypoxic training vs. the live high, train low strategy on aerobic capacity and sports performance in cyclists in normoxia. Biol Sport. 2018;35(1):39-48. Epub 2018/09/22. doi: 10.5114/biolsport.2018.70750. PubMed PMID: 30237660; PubMed Central PMCID: PMCPMC6135973.

67. Ambroży T, Maciejczyk M, Klimek AT, Wiecha S, Stanula A, Snopkowski P, et al. The Effects of Intermittent Hypoxic Training on Anaerobic and Aerobic Power in Boxers. Int J Environ Res Public Health. 2020;17(24). Epub 2020/12/18. doi: 10.3390/ijerph17249361. PubMed PMID: 33327551; PubMed Central PMCID: PMCPMC7765038.

68. Morton JP, Cable NT. Effects of intermittent hypoxic training on aerobic and anaerobic performance. Ergonomics. 2005;48(11-14):1535-46. Epub 2005/12/13. doi: 10.1080/00140130500100959. PubMed PMID: 16338719.

69. Czuba M, Bril G, Płoszczyca K, Piotrowicz Z, Chalimoniuk M, Roczniok R, et al. Intermittent Hypoxic Training at Lactate Threshold Intensity Improves Aiming Performance in Well-Trained Biathletes with Little Change of Cardiovascular Variables. Biomed Res Int. 2019;2019:1287506. Epub 2019/10/31. doi: 10.1155/2019/1287506. PubMed PMID: 31662969; PubMed Central PMCID: PMCPMC6778904 commercial or financial relationships that could be construed as potential conflicts of interest.

70. Kim SW, Jung WS, Kim JW, Nam SS, Park HY. Aerobic Continuous and Interval Training under Hypoxia Enhances Endurance Exercise Performance with Hemodynamic and Autonomic Nervous System Function in Amateur Male Swimmers. Int J Environ Res Public Health. 2021;18(8). Epub 2021/05/01. doi: 10.3390/ijerph18083944. PubMed PMID: 33918616; PubMed Central PMCID: PMCPMC8068973.

71. Dufour SP, Ponsot E, Zoll J, Doutreleau S, Lonsdorfer-Wolf E, Geny B, et al. Exercise training in normobaric hypoxia in endurance runners. I. Improvement in aerobic performance capacity. J Appl Physiol (1985). 2006;100(4):1238-48. Epub 2006/03/17. doi: 10.1152/japplphysiol.00742.2005. PubMed PMID: 16540709.

72. Sanchez AMJ, Borrani F. Effects of intermittent hypoxic training performed at high hypoxia level on exercise performance in highly trained runners. J Sports Sci. 2018;36(18):2045-52. Epub 2018/02/03. doi: 10.1080/02640414.2018.1434747. PubMed PMID: 29394148.

73. Robach P, Bonne T, Flück D, Bürgi S, Toigo M, Jacobs RA, et al. Hypoxic training: effect on mitochondrial function and aerobic performance in hypoxia. Med Sci Sports Exerc. 2014;46(10):1936-45. Epub 2014/03/29. doi: 10.1249/mss.0000000000000321. PubMed PMID: 24674976.

74. Hamlin MJ, Marshall HC, Hellemans J, Ainslie PN, Anglem N. Effect of intermittent hypoxic training on 20 km time trial and 30 s anaerobic performance. Scand J Med Sci Sports. 2010;20(4):651-61. Epub 2009/10/02. doi: 10.1111/j.1600-0838.2009.00946.x. PubMed PMID: 19793215.

75. Katayama K, Sato K, Matsuo H, Ishida K, Iwasaki K, Miyamura M. Effect of intermittent hypoxia on oxygen uptake during submaximal exercise in endurance athletes. Eur J Appl Physiol. 2004;92(1-2):75-83. Epub 2004/03/03. doi: 10.1007/s00421-004-1054-0. PubMed PMID: 14991325.

76. Park HY, Jung WS, Kim J, Hwang H, Lim K. Efficacy of intermittent hypoxic training on hemodynamic function and exercise performance in competitive swimmers. J Exerc Nutrition Biochem. 2018;22(4):32-8. Epub 2019/01/22. doi: 10.20463/jenb.2018.0028. PubMed PMID: 30661329; PubMed Central PMCID: PMCPMC6343766.

77. Rodríguez FA, Truijens MJ, Townsend NE, Stray-Gundersen J, Gore CJ, Levine BD. Performance of runners and swimmers after four weeks of intermittent hypobaric hypoxic exposure plus sea level training. J Appl Physiol (1985). 2007;103(5):1523-35. Epub 2007/08/11. doi: 10.1152/japplphysiol.01320.2006. PubMed PMID: 17690191.

78. Tadibi V, Dehnert C, Menold E, Bärtsch P. Unchanged anaerobic and aerobic performance after short-term intermittent hypoxia. Med Sci Sports Exerc. 2007;39(5):858-64. Epub 2007/05/01. doi: 10.1249/mss.0b013e31803349d9. PubMed PMID: 17468586.

79. Miller A, George K. The Effect of Intermittent Hypoxic Exposure plus Sea Level Swimming Training on Anaerobic Swimming Performance. Journal of Swimming Research. 2012;20.

80. Humberstone-Gough CE, Saunders PU, Bonetti DL, Stephens S, Bullock N, Anson JM, et al. Comparison of live high: train low altitude and intermittent hypoxic exposure. J Sports Sci Med. 2013;12(3):394-401. Epub 2013/10/24. PubMed PMID: 24149143; PubMed Central PMCID: PMCPMC3772580.

81. Burtscher M, Gatterer H, Faulhaber M, Gerstgrasser W, Schenk K. Effects of intermittent hypoxia on running economy. Int J Sports Med. 2010;31(9):644-50. Epub 2010/07/01. doi: 10.1055/s-0030-1255067. PubMed PMID: 20589591.

82. Hendriksen IJ, Meeuwsen T. The effect of intermittent training in hypobaric hypoxia on sea-level exercise: a cross-over study in humans. Eur J Appl Physiol. 2003;88(4-5):396-403. Epub 2003/01/16. doi: 10.1007/s00421-002-0708-z. PubMed PMID: 12527969.

83. Bonetti DL, Hopkins WG, Kilding AE. High-intensity kayak performance after adaptation to intermittent hypoxia. Int J Sports Physiol Perform. 2006;1(3):246-60. Epub 2006/09/01. doi: 10.1123/ijspp.1.3.246. PubMed PMID: 19116438.

84. Fernández-Lázaro D, Mielgo-Ayuso J, Santamaría G, Gutiérrez-Abejón E, Domínguez-Ortega C, García-Lázaro SM, et al. Adequacy of an Altitude Fitness Program (Living and Training) plus Intermittent Exposure to Hypoxia for Improving Hematological Biomarkers and Sports Performance of Elite Athletes: A Single-Blind Randomized Clinical Trial. Int J Environ Res Public Health. 2022;19(15). Epub 2022/07/29. doi: 10.3390/ijerph19159095. PubMed PMID: 35897470; PubMed Central PMCID: PMCPMC9368232.

85. Hamlin MJ, Hellemans J. Effect of intermittent normobaric hypoxic exposure at rest on haematological, physiological, and performance parameters in multi-sport athletes. J Sports Sci. 2007;25(4):431-41. Epub 2007/03/17. doi: 10.1080/02640410600718129. PubMed PMID: 17365530.
