# Supplementary material for: Effects of various living-low and training-high modes with distinct training prescriptions on sea-level performance: A network meta-analysis
Source: PLoS One. 2024 Apr 18;19(4):e0297007. doi: 10.1371/journal.pone.0297007 (PMC11025749; doi:10.1371/journal.pone.0297007)
Supplement: S1 File — (DOCX) [file pone.0297007.s005.docx]

**Supporting information file 2: Protocol**

Review title and timescale

1. Review title.

Give the title of the review in English

Effects of various living-low and training-high modes with distinct training prescriptions on sea-level performance: a network meta-analysis

2. Original language title.

For reviews in languages other than English, give the title in the original language. This will be displayed with the English language title.

3. Anticipated or actual start date.

Give the date the systematic review started or is expected to start.

30/6/2023

4. Anticipated completion date.

Give the date by which the review is expected to be completed.

15/9/2023

5. Stage of review at time of this submission.

Tick the boxes to show which review tasks have been started and which have been completed. Update this field each time any amendments are made to a published record.

Reviews that have started data extraction (at the time of initial submission) are not eligible for inclusion in PROSPERO. If there is later evidence that incorrect status and/or completion date has been supplied, the published PROSPERO record will be marked as retracted.

This field uses answers to initial screening questions. It cannot be edited until after registration.

The review has not yet started:

| Review stage | Started | Completed |
| --- | --- | --- |
| Preliminary searches | Yes | No |
| Piloting of the study selection process | Yes | No |
| Formal screening of search results against eligibility criteria | No | No |
| Data extraction | No | No |
| Risk of bias (quality) assessment | No | No |
| Data analysis | No | No |

Provide any other relevant information about the stage of the review here.

6. Named contact.

The named contact is the guarantor for the accuracy of the information in the register record. This may be any member of the review team.

Linlin Zhao

Email salutation (e.g. "Dr Smith" or "Joanne") for correspondence:

Professor Zhao

7. Named contact email.

Give the electronic email address of the named contact.

fengxinmiao666@163.com

8. Named contact address

Give the full institutional/organisational postal address for the named contact.

Xinxi Street, Haidian District, Beijing City, China

9. Named contact phone number.

Give the telephone number for the named contact, including international dialling code.

19933227661

10. Organisational affiliation of the review.

Full title of the organisational affiliations for this review and website address if available. This field may be completed as 'None' if the review is not affiliated to any organisation.

Sports coaching college, Beijing Sport University, 100084, Haidian, Beijing, China

Organisation web address:
11. Review team members and their organisational affiliations.

Give the personal details and the organisational affiliations of each member of the review team. Affiliation refers to groups or organisations to which review team members belong. **NOTE: email and country now MUST be entered for each person, unless you are amending a published record.**

Dr Xinmiao Feng. Sports coaching college, Beijing Sport University, 100084, Haidian, Beijing, China

Dr Yonghui Chen. Sports coaching college, Beijing Sport University, 100084, Haidian, Beijing, China

Dr Tieshuai Yan. Sports coaching college, Beijing Sport University, 100084, Haidian, Beijing, China

Dr Chuangang Wang. Sports coaching college, Beijing Sport University, 100084, Haidian, Beijing, China

Professor Linlin Zhao. Sports coaching college, Beijing Sport University, 100084, Haidian, Beijing, China

12. Funding sources/sponsors.

Details of the individuals, organizations, groups, companies or other legal entities who have funded or sponsored the review.

None

13. Conflicts of interest.

List actual or perceived conflicts of interest (financial or academic).

None

14. Collaborators.

Give the name and affiliation of any individuals or organisations who are working on the review but who are not listed as review team members. NOTE: email and country must be completed for each person, unless you are amending a published record.

15. Review question.

State the review question(s) clearly and precisely. It may be appropriate to break very broad questions down into a series of related more specific questions. Questions may be framed or refined using PI(E)COS or similar where relevant.

1) Are hypoxic training more effective than normoxic training for anaerobic and aerobic performance in athletes?
2) Are there specific hypoxic training (types, intensity, volume) that are more effective than others for anaerobic and aerobic performance in athletes?

16. Searches.

State the sources that will be searched (e.g. Medline). Give the search dates, and any restrictions (e.g. language or publication date). Do NOT enter the full search strategy (it may be provided as a link or attachment below.)

The systematic review and meta-analysis were conducted in accordance with the Preferred Reporting Items for Systematic Reviews and Meta-Analyses (PRISMA) guidelines. We will search PubMed, Web of Science, Embase, EBSCO, and Cochrane (Cochrane Database of Systematic Reviews, Cochrane Central Register of Controlled Trials (CENTRAL), Cochrane Methodology Register).

Studies published between inception and the date the searches are run will be sought. The searches will be re-run just before the final analyses and further studies retrieved for inclusion. We will use a Boolean search strategy with the operators AND, OR, NOT, and the search strategy will include terms describing or relating to intervention, participants, and study design.

17. URL to search strategy.

Upload a file with your search strategy, or an example of a search strategy for a specific database, (including the keywords) in pdf or word format. In doing so you are consenting to the file being made publicly accessible. Or provide a URL or link to the strategy. Do NOT provide links to your search results.

I give permission for this file to be made publicly available

Yes

18. Condition or domain being studied.

Give a short description of the disease, condition or healthcare domain being studied in your systematic review.

hypoxic training for athletes

19. Participants/population.

Specify the participants or populations being studied in the review. The preferred format includes details of both inclusion and exclusion criteria.

Age: adult ('18+')

Sex: Males and females

Race: Any

Athletes

20. Intervention(s), exposure(s).

Give full and clear descriptions or definitions of the interventions or the exposures to be reviewed. The preferred format includes details of both inclusion and exclusion criteria.

The intervention to be reviewed is: hypoxic training, including
1) intermittent hypoxic exposure;

2) continuous hypoxic training;

3) repeated sprint training in hypoxia;

4) interval sprint training in hypoxia;

5) intermittent hypoxic training;

7) continuous and interval training in hypoxia.

21. Comparator(s)/control.

Where relevant, give details of the alternatives against which the intervention/exposure will be compared (e.g. another intervention or a non-exposed control group). The preferred format includes details of both inclusion and exclusion criteria.

Control group: normoxic training

22. Types of study to be included.

Give details of the study designs (e.g. RCT) that are eligible for inclusion in the review. The preferred format includes both inclusion and exclusion criteria. If there are no restrictions on the types of study, this should be stated.

Include only randomized controlled trials

23. Context.

Give summary details of the setting or other relevant characteristics, which help define the inclusion or exclusion criteria.

24. Main outcome(s).

Give the pre-specified main (most important) outcomes of the review, including details of how the outcome is

1. Aerobic performance
2. Anaerobic performance

Measures of effect

Please specify the effect measure(s) for you main outcome(s) e.g. relative risks, odds ratios, risk difference, and/or 'number needed to treat.

Timing: The specified end point of the trial will be used as the end-point in the assessment.

Effect measure: Standardised mean difference at the end of the study

25. Additional outcome(s).

List the pre-specified additional outcomes of the review, with a similar level of detail to that required for main outcomes. Where there are no additional outcomes please state ‘None’ or ‘Not applicable’ as appropriate to the review

None

Measures of effect

Please specify the effect measure(s) for you additional outcome(s) e.g. relative risks, odds ratios, risk difference, and/or 'number needed to treat.

26. Data extraction (selection and coding).

Describe how studies will be selected for inclusion. State what data will be extracted or obtained. State how this will be done and recorded.

All search results will be exported into EndNote and duplicates will be removed. Titles and abstracts from the initial literature search will be independently assessed by two reviewers (X.M.F and L.L.Z). Full texts for articles deemed eligible for inclusion from the title and abstract search by either reviewer, in addition to those where no decision could be reached by the reviewers from this initial screen, will be screened independently by two researchers (X.M.F and H.Y.L). Any discrepancies will be resolved by discussion with all researchers in the review team. Two reviewers (X.M.F and Y.H.C) will independently extract data from the final inclusion list of articles into a standardised data extraction spreadsheet in Excel. At this stage, two authors extracted information on (1) relevant data regarding participant characteristics (e.g., the sample size, age, and sex); (2) training pattern; (3) training variable (e.g., duration, frequency, sets, repetitions, and intensity); (4) Sports that the athletes participate in; (5) the main result of the study. In case of incomplete raw data availability, we contacted the corresponding author of the manuscript. We excluded the studies of which the authors could not be reached. All studies were assessed independently in this systematic review by two researchers (X.M.F. and T.S.Y) based on the extracted information. If there were any disagreements about the inclusion of a study, a third reviewer (L.L.Z) was consulted.

27. Risk of bias (quality) assessment.

State which characteristics of the studies will be assessed and/or any formal risk of bias/quality assessment tools that will be used.

The study quality was assessed with the PEDro scale, based on the list of Delphi (Verhagen et al., 1998). The PEDro scale includes 11 items with three items from the Jadad scale (Jadad et al., 1996) and nine items from the Delphi list (Verhagen et al., 1998). PEDro rates RCTs on a scale from 0 (low quality) to 10 (high quality), and scores less than 6 are considered to have low methodological quality, as per the PEDro database statistics (Maher, Sherrington, Herbert, Moseley, & Elkins, 2003). Interrater reliability was shown to be fair to good (Intraclass Correlation Coefficient = 0.68). Two reviewers (X.M.F and L.L.Z) scored the studies according to the proposed scale. In case of disagreements, a consensus was adopted or, if necessary, a third reviewer evaluated the article (Y.H.C).

28. Strategy for data synthesis.

Describe the methods you plan to use to synthesise data. This must not be generic text but should be specific to your review and describe how the proposed approach will be applied to your data.
If meta-analysis is planned, describe the models to be used, methods to explore statistical heterogeneity, and software package to be used.

Network meta-analysis is planned for the multiple treatment comparison. Heterogeneity will be assessed, in addition to consistency which will be examined by fitting consistency and inconsistency models. This will determine if the treatment effect for a pair of treatments estimated from an indirect comparison is consistent with the treatment effect estimated from a direct comparison.

29. Analysis of subgroups or subsets.

State any planned investigation of ‘subgroups’. Be clear and specific about which type of study or participant will be included in each group or covariate investigated. State the planned analytic approach.

None

30. Type and method of review.

Select the type of review, review method and health area from the lists below.

Network meta-analysis

31. Language.

Select each language individually to add it to the list below, use the bin icon to remove any added in error.

English

32. Country.

Select the country in which the review is being carried out. For multi-national collaborations select all the countries involved.

China

33. Other registration details.

Name any other organisation where the systematic review title or protocol is registered (e.g. Campbell, or The Joanna Briggs Institute) together with any unique identification number assigned by them. If extracted data will be stored and made available through a repository such as the Systematic Review Data Repository (SRDR), details and a link should be included here. If none, leave blank.

34. Reference and/or URL for published protocol.

If the protocol for this review is published provide details (authors, title and journal details, preferably in Vancouver format)

I give permission for this file to be made publicly available

Yes

35  Dissemination plans

Give brief details of plans for communicating essential messages from the review to the appropriate audiences.

Do you intend to publish the review on completion?

Yes

36  Keywords
Give words or phrases that best describe the review. (One word per box, create a new box for each term)

Key words: hypoxic training; aerobic performance; anaerobic performance; athletes; network meta-analysis

37. Details of any existing review of the same topic by the same authors.

If you are registering an update of an existing review give details of the earlier versions and include a full bibliographic reference, if available.

38. Current review status.

Update review status when the review is completed and when it is published.
New registrations must be ongoing so this field is not editable for initial submission.

Ongoing

39. Any additional information.

Provide any other information relevant to the registration of this review.

None

40. Details of final report/publication(s) or preprints if available.

Leave empty until publication details are available OR you have a link to a preprint (NOTE: this field is not editable for initial submission).
List authors, title and journal details preferably in Vancouver format.

Give the link to the published review or preprint.
