# Supplementary material for: Effects of various living-low and training-high modes with distinct training prescriptions on sea-level performance: A network meta-analysis
Source: PLoS One. 2024 Apr 18;19(4):e0297007. doi: 10.1371/journal.pone.0297007 (PMC11025749; doi:10.1371/journal.pone.0297007)
Supplement: S2 File — (DOCX) [file pone.0297007.s006.docx]

**Supporting information 4: classification and definition of various LLTH modes**

**Figure 1. Classification of LLTH modes**

**
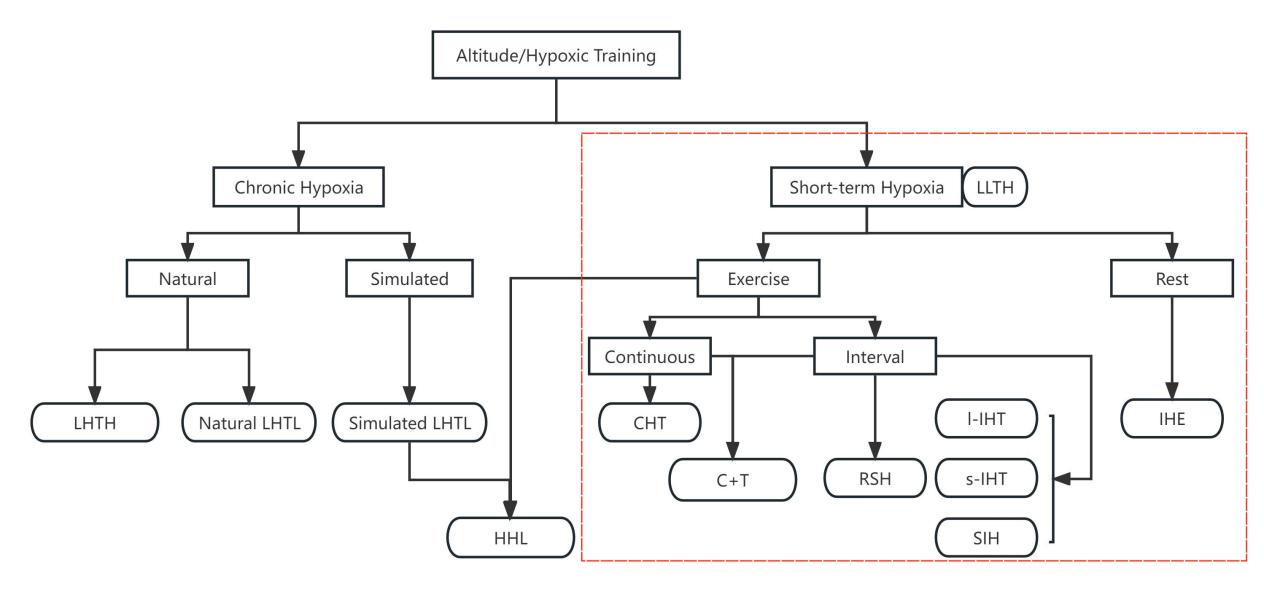
**

Figure 1. classification of LLTH modes. LHTH: living-high and training-high; LHTL: living-high and training-low; LLTH: living-low and training-high; HHL: living-high and training-high and-low; CHT: continuous hypoxic training; IHE: intermittent Hypoxic exposure; RSH: repeated sprint training in hypoxia; ISH: interval sprint training in hypoxia; l-IHT: long-durations interval hypoxic training; s-IHT: short-durations interval hypoxic training; C + T: continuous and interval training under Hypoxia.

**Table 1. Definition of LLTH modes**

| Hypoxic training mode | Definition |
| --- | --- |
| Repeated sprint training in hypoxia  (RSH) | the repetition of several short “all-out” exercise bouts (≤15 s) in hypoxia interspersed with incomplete recoveries (30 s,exercise-to-rest ratio <1:4) |
| Interval sprint  training in hypoxia  (ISH) | Several “all-out” sprints bouts (usually 30 s) in hypoxia interspersed with recoveries (2-5 min) |
| Short-duration  high-intensity  Interval training  (s-IHT) | Several short-term high-intensity exercise bouts (1-3 min) in hypoxia interspersed with recoveries (2-5min) |
| Long-duration  high-intensity  interval training  (l-IHT) | Several long-term high-intensity exercise bouts (＞3 min) in hypoxia interspersed with recoveries (2-5min) |
| Continuous  hypoxic training  (CHT) | Moderate-high intensity continuous training (30-60min) in hypoxia |
| Continuous and Interval training under Hypoxia  (C+I) | 1. One session consisting of continuous and interval training 2. Interval and continuous training sessions were conducted separately during a week |
| Intermittent  Hypoxic exposure  (IHE) | intermittent exposure to a severe hypoxia during rest   1. Alternatively receiving normoxia and hypoxia exposure 2. Persistently receiving hypoxia exposure |

Table 1. Definition of LLTH modes
