# Supplementary material for: Effects of various living-low and training-high modes with distinct training prescriptions on sea-level performance: A network meta-analysis
Source: PLoS One. 2024 Apr 18;19(4):e0297007. doi: 10.1371/journal.pone.0297007 (PMC11025749; doi:10.1371/journal.pone.0297007)
Supplement: S3 File — (DOCX) [file pone.0297007.s007.docx]

**Supporting information file 5: hypoxic dose model**

The hypoxic dosage between different hypoxic types was coordinated using the "kilometer hours" model [1]. The dosage model was defined as km·h = (m/1000)× h (“m” represents the altitude of the exposure environment; “h” represents the total exposure duration).

1. Garvican-Lewis LA, Sharpe K, Gore CJ. Time for a new metric for hypoxic dose? J Appl Physiol (1985). 2016;121(1):352-5. Epub 2016/02/27. doi: 10.1152/japplphysiol.00579.2015. PubMed PMID: 26917695.
