# Supplementary material for: Effects of various living-low and training-high modes with distinct training prescriptions on sea-level performance: A network meta-analysis
Source: PLoS One. 2024 Apr 18;19(4):e0297007. doi: 10.1371/journal.pone.0297007 (PMC11025749; doi:10.1371/journal.pone.0297007)
Supplement: S4 File — (DOCX) [file pone.0297007.s008.docx]

**Supporting information table 6: selection of reference indicators**

| Reference indicator of aerobic performance | |
| --- | --- |
| *Selected test* | *Selected results* |
| incremental treadmill test  [2-6] | PPO(W);  TTE(s); MAS(km·h-1) |
| 3 min All-Out Test  [7, 8] | APO(W) |
| YYIR test level I and II  [9-12] | Ddistance Covered(m); |
| *Field test：*  Run test (distance≥1000m) and  [13-17]  Cycle test (durations≥10min)  [18]  Swiming test (distance≥400m)  [19] | Duration(s/min) |
| constant-load test | TTE(s) |
| Reference indicator of unaerobic performance | |
| *Selected test* | *Selected results* |
| RAS test  [20] | PPO(W) |
| Maximal anaerobic test; Supramaximal time to fatigue test  [21-23] | Duration(s) |
| Wingate Test  [23-25] | PPO(W)  APO(W) |
| *Field test：*  Run test (distance≤800m/durations≤2min); Sprint test  [26-28]  Swiming test (≤200m)  [29, 30] | Duration(s) |

Table 2. Selection of reference indicators. PPO: peak power output; APO: average power output; TTE: Time to exhaustion; MAS: Maximal aerobic speed; RSA test: repeated sprint ability test.

2. Billat LV, Koralsztein JP. Significance of the velocity at VO2max and time to exhaustion at this velocity. Sports Med. 1996;22(2):90-108. Epub 1996/08/01. doi: 10.2165/00007256-199622020-00004. PubMed PMID: 8857705.

3. Hopkins WG, Schabort EJ, Hawley JA. Reliability of power in physical performance tests. Sports Med. 2001;31(3):211-34. Epub 2001/04/05. doi: 10.2165/00007256-200131030-00005. PubMed PMID: 11286357.

4. Midgley AW, Bentley DJ, Luttikholt H, McNaughton LR, Millet GP. Challenging a dogma of exercise physiology: does an incremental exercise test for valid VO 2 max determination really need to last between 8 and 12 minutes? Sports Med. 2008;38(6):441-7. Epub 2008/05/21. doi: 10.2165/00007256-200838060-00001. PubMed PMID: 18489192.

5. Kuipers H, Rietjens G, Verstappen F, Schoenmakers H, Hofman G. Effects of stage duration in incremental running tests on physiological variables. Int J Sports Med. 2003;24(7):486-91. Epub 2003/09/12. doi: 10.1055/s-2003-42020. PubMed PMID: 12968205.

6. Kang J, Chaloupka EC, Mastrangelo MA, Biren GB, Robertson RJ. Physiological comparisons among three maximal treadmill exercise protocols in trained and untrained individuals. Eur J Appl Physiol. 2001;84(4):291-5. Epub 2001/05/26. doi: 10.1007/s004210000366. PubMed PMID: 11374112.

7. Burnley M, Doust JH, Vanhatalo A. A 3-min all-out test to determine peak oxygen uptake and the maximal steady state. Med Sci Sports Exerc. 2006;38(11):1995-2003. Epub 2006/11/11. doi: 10.1249/01.mss.0000232024.06114.a6. PubMed PMID: 17095935.

8. Kramer M, Du Randt R, Watson M, Pettitt RW. Energetics of male field-sport athletes during the 3-min all-out test for linear and shuttle-based running. Eur J Appl Physiol. 2019;119(2):477-86. Epub 2018/12/05. doi: 10.1007/s00421-018-4047-0. PubMed PMID: 30511278.

9. Krustrup P, Mohr M, Amstrup T, Rysgaard T, Johansen J, Steensberg A, et al. The yo-yo intermittent recovery test: physiological response, reliability, and validity. Med Sci Sports Exerc. 2003;35(4):697-705. Epub 2003/04/04. doi: 10.1249/01.Mss.0000058441.94520.32. PubMed PMID: 12673156.

10. Vanhatalo A, Doust JH, Burnley M. A 3-min all-out cycling test is sensitive to a change in critical power. Med Sci Sports Exerc. 2008;40(9):1693-9. Epub 2008/08/08. doi: 10.1249/MSS.0b013e318177871a. PubMed PMID: 18685519.

11. Grgic J, Oppici L, Mikulic P, Bangsbo J, Krustrup P, Pedisic Z. Test-Retest Reliability of the Yo-Yo Test: A Systematic Review. Sports Med. 2019;49(10):1547-57. Epub 2019/07/05. doi: 10.1007/s40279-019-01143-4. PubMed PMID: 31270753.

12. Bangsbo J, Iaia FM, Krustrup P. The Yo-Yo intermittent recovery test : a useful tool for evaluation of physical performance in intermittent sports. Sports Med. 2008;38(1):37-51. Epub 2007/12/18. doi: 10.2165/00007256-200838010-00004. PubMed PMID: 18081366.

13. Gaesser GA, Brooks GA. Metabolic bases of excess post-exercise oxygen consumption: a review. Med Sci Sports Exerc. 1984;16(1):29-43. Epub 1984/01/01. PubMed PMID: 6369064.

14. Green HJ, Hughson RL. Anaerobic threshold: review of the concept and directions for future research. Med Sci Sports Exerc. 1985;17(5):621-4. Epub 1985/10/01. PubMed PMID: 4068970.

15. Gastin PB. Energy system interaction and relative contribution during maximal exercise. Sports Med. 2001;31(10):725-41. Epub 2001/09/08. doi: 10.2165/00007256-200131100-00003. PubMed PMID: 11547894.

16. Jones AM, Carter H. The effect of endurance training on parameters of aerobic fitness. Sports Med. 2000;29(6):373-86. Epub 2000/06/28. doi: 10.2165/00007256-200029060-00001. PubMed PMID: 10870864.

17. Brandon LJ. Physiological factors associated with middle distance running performance. Sports Med. 1995;19(4):268-77. Epub 1995/04/01. doi: 10.2165/00007256-199519040-00004. PubMed PMID: 7604199.

18. Abbiss CR, Laursen PB. Models to explain fatigue during prolonged endurance cycling. Sports Med. 2005;35(10):865-98. Epub 2005/09/27. doi: 10.2165/00007256-200535100-00004. PubMed PMID: 16180946.

19. Toussaint HM, Hollander AP. Energetics of Competitive Swimming. Sports Medicine. 1994;18(6):384.

20. Girard O, Mendez-Villanueva A, Bishop D. Repeated-sprint ability - part I: factors contributing to fatigue. Sports Med. 2011;41(8):673-94. Epub 2011/07/26. doi: 10.2165/11590550-000000000-00000. PubMed PMID: 21780851.

21. Rusko HK, Nummela A. Measurement of maximal and submaximal anaerobic performance capacity: concluding chapter. Int J Sports Med. 1996;17 Suppl 2:S125-9. Epub 1996/07/01. doi: 10.1055/s-2007-972913. PubMed PMID: 8844277.

22. Katch V, Weltman A, Martin R, Gray L. Optimal test characteristics for maximal anaerobic work on the bicycle ergometer. Res Q. 1977;48(2):319-27. Epub 1977/05/01. PubMed PMID: 267972.

23. Vandewalle DH, Péerès G, Monod H. Standard Anaerobic Exercise Tests. Sports Medicine. 1987;4(4):268.

24. Calbet JA, Chavarren J, Dorado C. Fractional use of anaerobic capacity during a 30- and a 45-s Wingate test. Eur J Appl Physiol Occup Physiol. 1997;76(4):308-13. Epub 1997/01/01. doi: 10.1007/s004210050253. PubMed PMID: 9349644.

25. Granier P, Mercier B, Mercier J, Anselme F, Préfaut C. Aerobic and anaerobic contribution to Wingate test performance in sprint and middle-distance runners. Eur J Appl Physiol Occup Physiol. 1995;70(1):58-65. Epub 1995/01/01. doi: 10.1007/bf00601809. PubMed PMID: 7729439.

26. Hautier CA, Wouassi D, Arsac LM, Bitanga E, Thiriet P, Lacour JR. Relationships between postcompetition blood lactate concentration and average running velocity over 100-m and 200-m races. Eur J Appl Physiol Occup Physiol. 1994;68(6):508-13. Epub 1994/01/01. doi: 10.1007/bf00599521. PubMed PMID: 7957143.

27. Zamparo P, Bolomini F, Nardello F, Beato M. Energetics (and kinematics) of short shuttle runs. Eur J Appl Physiol. 2015;115(9):1985-94. Epub 2015/05/13. doi: 10.1007/s00421-015-3180-2. PubMed PMID: 25963378.

28. di Prampero PE, Fusi S, Sepulcri L, Morin JB, Belli A, Antonutto G. Sprint running: a new energetic approach. J Exp Biol. 2005;208(Pt 14):2809-16. Epub 2005/07/08. doi: 10.1242/jeb.01700. PubMed PMID: 16000549.

29. Toussaint HM, Hollander AP. Energetics of competitive swimming. Implications for training programmes. Sports Med. 1994;18(6):384-405. Epub 1994/12/01. doi: 10.2165/00007256-199418060-00004. PubMed PMID: 7886354.

30. Figueiredo P, Barbosa TM, Vilas-Boas JP, Fernandes RJ. Energy cost and body centre of mass' 3D intracycle velocity variation in swimming. Eur J Appl Physiol. 2012;112(9):3319-26. Epub 2012/01/21. doi: 10.1007/s00421-011-2284-6. PubMed PMID: 22262010.
