# Supplementary material for: Effects of various living-low and training-high modes with distinct training prescriptions on sea-level performance: A network meta-analysis
Source: PLoS One. 2024 Apr 18;19(4):e0297007. doi: 10.1371/journal.pone.0297007 (PMC11025749; doi:10.1371/journal.pone.0297007)
Supplement: S5 File — (DOCX) [file pone.0297007.s009.docx]

**Supporting information file 9: Statistical methods in details**

## Network meta-analysis

We will use R software gemtc and rjags packages to perform Bayesian network meta-analysis. Using arm-level data and import into the R software in CSV format. The effect size measure for continuous outcomes chooses the standardized mean difference (SMD) of the change score (end-point minus baseline score) because the studies use aerobic and anaerobic manifestations. The normal likelihood for continuous outcomes. The study effect sizes were then synthesized using a random-effects network meta-analysis model. In addition, we will present the summary SMD, 95% credible intervals (CrIs) for all pairwise comparisons in the league table, and we show the results of comparing the outcomes of each hypoxia training group and the normoxia training group in the form of a forest plot. To rank the various interventions for each outcome, we will use the surface under the cumulative ranking curve (SUCRA). In the process of extracting data, if the original study reported a standard error in the experimental and control groups, the standard deviation was calculated by the formula: standard deviation (SD) = standard error (SE) × √n. If both are missing, we will estimate SD based on the confidence interval, t-value, quartile, range, or p-values as described in section 7.7.3 of the Cochrane Handbook for Systematic Reviews. When only figures were presented, data were extracted using GetData (http://getdata-graph-digitizer.com) to measure the length (in pixels) of the axes to calibrate and then the length in pixels from the relevant axis to the data points of interest. If the data needed for the study cannot be extracted from the above methods, we will ask the authors about the data at least 4 times within 6 weeks.

## Continuous variable-random effects model:

For any hypoxia training modes x in any randomized controlled experiment i, the sample size is n_i, x_. The effect of interventions is y_i, x_ (change from baseline), and standard error is se_i, x_. Then the normal

likelihood is employed to y_i, x_ ~ N (𝜃_i, y_, se_i, x_) in each arm. In addition, for any randomized controlled experiment, there should be a basic hypoxia training mode b(i), and its effect is represented by y_i, b(i)_

y_i, b(i)_ = 𝑢_𝑖_

In the random effects model, for any interventional physical activity k that is not a basic hypoxia training mode, its training effect is:

𝜃_i, k_= 𝑢_𝑖_+𝛿_i, b(i), k_ for k ≥ 2

Where 𝛿_i, b(i), k_ is the difference between the effect of hypoxia training k and normoxia training b, and conforms to the following normal distribution 𝛿_i, x, y_ ~ N(d_x,y_, 𝞼^2^_x, y_), where d_x,y_ is the relative effect of hypoxia training intervention y and x, 𝞼^2^_x, y_ is the variance of the relative effect of hypoxia training intervention y and x. In addition, this study presented the final training effect with standardised mean difference (SMD), so the above formula is modified:

y_i, b(i)_ = 𝑢_𝑖_/S_i_

𝜃_i, k_= (𝑢_𝑖_+𝛿_i, b(i), y_)/ S_i_ for k ≥ 2

where S_i_ is the pooled standard deviation in the study arms.

model {

# Likelihood for arm-based data

## OMITTED

# Likelihood for contrast-based data (univariate for 2-arm trials)

for(i in studies.r2) {

for (k in 2:na[i]) {

mest[i, k] <- delta[i, k]

}

m[i, 2] ~ dnorm(mest[i, 2], prec[i, 2])

prec[i, 2] <- 1 / (e[i, 2] * e[i, 2])

dev[i, 1] <- pow(m[i, 2] - mest[i, 2], 2) * prec[i, 2]

}

# Likelihood for contrast-based data (multivariate for multi-arm trials)

for(i in studies.rm) {

for (k in 2:na[i]) {

mest[i, k] <- delta[i, k]

}

for (k in 1:(na[i]-1)) {

for (j in 1:(na[i]-1)) {

Sigma[i,j,k] <- ifelse(equals(j, k), pow(e[i,k+1], 2), pow(e[i,1], 2))

}

}

Omega[i,1:(na[i]-1),1:(na[i]-1)] <- inverse(Sigma[i,1:(na[i]-1),1:(na[i]-1)])

m[i,2:na[i]] ~ dmnorm(mest[i,2:na[i]], Omega[i,1:(na[i]-1),1:(na[i]-1)])

mdiff[i, 2:na[i]] <- m[i, 2:na[i]] - mest[i, 2:na[i]]

dev[i, 1] <- t(mdiff[i, 2:na[i]]) %*% Omega[i, 1:(na[i]-1),1:(na[i]-1)] %*% mdiff[i, 2:na[i]]

}

# Random effects model

for (i in studies) {

# Study-level relative effects

w[i, 1] <- 0

delta[i, 1] <- 0

for (k in 2:na[i]) { # parameterize multi-arm trials using a trick to avoid dmnorm

delta[i, k] ~ dnorm(md[i, k], taud[i, k])

md[i, k] <- d[t[i, 1], t[i, k]] + sw[i, k]

taud[i, k] <- tau.d * 2 * (k - 1) / k

w[i, k] <- delta[i, k] - (d[t[i, 1], t[i, k]])

sw[i, k] <- sum(w[i, 1:(k-1)]) / (k - 1)

}

}

# Random effects variance prior

sd.d ~ dunif(0, om.scale)

tau.d <- pow(sd.d, -2)

# Relative effect matrix

d[1, 1] <- 0

d[1, 2] <- d.AE.RSH

d[1, 3] <- d.AE.ISH

d[1, 4] <- d.AE.IHE

d[1, 5] <- d.AE.CHT

d[1, 6] <- d.AE.s-IHT

d[1, 7] <- d.AE.l-IHT

d[1, 8] <- d.AE.C+I

d[1, 9] <- d.AE.CON

for (i in 2:nt) {

for (j in 1:nt) {

d[i, j] <- d[1, j] - d[1, i]

}

}

prior.prec <- pow(re.prior.sd, -2)

# Study baseline priors

## OMITTED

# Effect parameter priors

d.RSH ~ dnorm(0, prior.prec)

d.ISH ~ dnorm(0, prior.prec)

d.IHE ~ dnorm(0, prior.prec)

d.CHT ~ dnorm(0, prior.prec)

d.s-IHT ~ dnorm(0, prior.prec)

d.l-IHT ~ dnorm(0, prior.prec)

d.C+I(0, prior.prec)

d.CON(0, prior.prec)

}

## Network meta-regression: In the network meta-regression models we set

𝜃 𝑖_,𝑘_ =𝜃 +𝛽_1,𝜅_ ×(𝑥 _𝑖_ −𝑟𝑒𝑓 _𝑥_)

In the model with independent and consistent coefficients we define 𝛽_1,k_ = 𝛽_k_. if treatment 1 is control group (normoxia training; otherwise 𝛽_1, k_ ≡ 𝛽_AB_ = 𝛽_B_ − 𝛽_A_ for any interventions 𝐴, 𝐵. In the model with exchangeable coefficients we set 𝛽_K_ ~𝑁(𝛣, 𝜏^2^_B_ ).

model {

# Likelihood for arm-based data

## OMITTED

# Likelihood for contrast-based data (univariate for 2-arm trials)

for(i in studies.r2) {

for (k in 2:na[i]) {

mest[i, k] <- delta[i, k] + (beta[t[i, k]] - beta[t[i, 1]]) * x[i]

}

m[i, 2] ~ dnorm(mest[i, 2], prec[i, 2])

prec[i, 2] <- 1 / (e[i, 2] * e[i, 2])

dev[i, 1] <- pow(m[i, 2] - mest[i, 2], 2) * prec[i, 2]

}

# Likelihood for contrast-based data (multivariate for multi-arm trials)

for(i in studies.rm) {

for (k in 2:na[i]) {

mest[i, k] <- delta[i, k] + (beta[t[i, k]] - beta[t[i, 1]]) * x[i]

}

for (k in 1:(na[i]-1)) {

for (j in 1:(na[i]-1)) {

Sigma[i,j,k] <- ifelse(equals(j, k), pow(e[i,k+1], 2), pow(e[i,1], 2))

}

}

Omega[i,1:(na[i]-1),1:(na[i]-1)] <- inverse(Sigma[i,1:(na[i]-1),1:(na[i]-1)])

m[i,2:na[i]] ~ dmnorm(mest[i,2:na[i]], Omega[i,1:(na[i]-1),1:(na[i]-1)])

mdiff[i, 2:na[i]] <- m[i, 2:na[i]] - mest[i, 2:na[i]]

dev[i, 1] <- t(mdiff[i, 2:na[i]]) %*% Omega[i, 1:(na[i]-1),1:(na[i]-1)] %*% mdiff[i, 2:na[i]]

}

# Random effects model

for (i in studies) {

# Study-level relative effects

w[i, 1] <- 0

delta[i, 1] <- 0

for (k in 2:na[i]) { # parameterize multi-arm trials using a trick to avoid dmnorm

delta[i, k] ~ dnorm(md[i, k], taud[i, k])

md[i, k] <- d[t[i, 1], t[i, k]] + sw[i, k]

taud[i, k] <- tau.d * 2 * (k - 1) / k

w[i, k] <- delta[i, k] - (d[t[i, 1], t[i, k]])

sw[i, k] <- sum(w[i, 1:(k-1)]) / (k - 1)

}

}

# Random effects variance prior

sd.d ~ dunif(0, om.scale)

tau.d <- pow(sd.d, -2)

# Relative effect matrix

d[1, 1] <- 0

d[1, 2] <- d.AE.RSH

d[1, 3] <- d.AE.ISH

d[1, 4] <- d.AE.IHE

d[1, 5] <- d.AE.CHT

d[1, 6] <- d.AE.l-IHT

d[1, 7] <- d.AE.s-IHT

d[1, 8] <- d.AE.C+I

d[1, 9] <- d.AE.CON

for (i in 2:nt) {

for (j in 1:nt) {

d[i, j] <- d[1, j] - d[1, i]

}

}

prior.prec <- pow(re.prior.sd, -2)

# Study baseline priors

## OMITTED

# Effect parameter priors

d.AE.RSH ~ dnorm(0, prior.prec)

d.AE.ISH ~ dnorm(0, prior.prec)

d.AE.IHE ~ dnorm(0, prior.prec)

d.AE.CHT ~ dnorm(0, prior.prec)

d.AE.s-IHT ~ dnorm(0, prior.prec)

d.AE.l-IHT ~ dnorm(0, prior.prec)

d.AE.C+I ~ dnorm(0, prior.prec)

# Regression priors

reg.prior.prec <- pow(om.scale, -2)

for (k in c(1:(reg.control-1), (reg.control+1):nt)) {

beta[k] <- B

}

beta[reg.control] <- 0

B ~ dt(0, reg.prior.prec, 1)

## Assessment of the transitivity assumption

None

## Assessment of heterogeneity and inconsistency

We use the tau square (τ^2^) test and p-value to qualitatively analyze the statistical heterogeneity between the studies. The larger the τ^2^ and the smaller the p-value, the greater the possibility of heterogeneity; on the contrary, the smaller the existence heterogeneity. In addition, I^2^ is a parameter for quantitative analysis of the heterogeneity between the results of each study. It’ s value is distributed from 0-100%. When I^2^ is less than 25%, it means that the heterogeneity is low; 25%-50% means that the heterogeneity is moderate; I^2^ > 75% means high heterogeneity. In summary, when I^2^ > 50%, it means that there is substantial heterogeneity. We will use global and local methods to test the inconsistency of the research results. For global inconsistency, we evaluated inconsistency statistically using the design-by-treatment test. In addition, we will assessment of local inconsistency by separating indirect from direct evidence (SIDE test) using the R netmeta package.

## Publication bias

We compared the adjusted funnel plot to assess the risk of publication bias under specific circumstances. In addition, we made a linear fit for the primary outcome. When the fitted line coincides with the 0 quadrant, it means that there is no published bias. For multi-arm trails, for example, hypoxic training type A, B and control, we plotted control group vs hypoxic training type A, and control group and hypoxic training type B

## Assessment of sensitivity

None
