# Supplementary material for: Effects of various living-low and training-high modes with distinct training prescriptions on sea-level performance: A network meta-analysis
Source: PLoS One. 2024 Apr 18;19(4):e0297007. doi: 10.1371/journal.pone.0297007 (PMC11025749; doi:10.1371/journal.pone.0297007)
Supplement: S6 File — (DOCX) [file pone.0297007.s010.docx]

# Supporting information file 10: Evaluation of heterogeneity and inconsistency

## 9.1 Quantifying heterogeneity

We use the tau square (τ^2^) test and p-value to qualitatively analyze the statistical heterogeneity between the studies. The larger the τ^2^ and the smaller the p-value, the greater the possibility of heterogeneity; on the contrary, the smaller the existence heterogeneity. In addition, I^2^ is a parameter for quantitative analysis of the heterogeneity between the results of each study. It’ s value is distributed from 0-100%. When I^2^ is less than 25%, it means that the heterogeneity is low; 25%-50% means that the heterogeneity is moderate; I^2^ > 75% means high heterogeneity. In summary, when I^2^ > 50%, it means that there is substantial heterogeneity.

| Primary outcomes | τ^2^ | Q | df | P | I^2^ | Heterogeneity assessment |
| --- | --- | --- | --- | --- | --- | --- |
| Aerobic performance | 0.0206 | 78.29 | 72 | 0.2860 | 8.0% | low |
| Anaerobic performance | 0.0585 | 60.37 | 48 | 0.1085 | 20.5% | low |

## 9.2 Evaluation of inconsistency

**Summary of the global inconsistency and SIDE splitting results**

| Outcomes | Number of studies | the Design-by-Treatment test | | | |
| --- | --- | --- | --- | --- | --- |
|  |  | Q | df | τ^2^ | p-value |
| Aerobic performance | 53 | 1.52 | 3 | 0.1608 | 0.6774 |
| Anaerobic performance | 29 | 0.14 | 1 | 0.0634 | 0.7037 |
