# Supplementary material for: Effects of various living-low and training-high modes with distinct training prescriptions on sea-level performance: A network meta-analysis
Source: PLoS One. 2024 Apr 18;19(4):e0297007. doi: 10.1371/journal.pone.0297007 (PMC11025749; doi:10.1371/journal.pone.0297007)
Supplement: Attachment — Submitted filename: Response to Reviewers.docx [file pone.0297007.s012.docx]

**Response to Reviewers**

Dear Reviewers,

I sincerely appreciate the time and effort you have taken to review and provide insightful feedback on my manuscript titled “[Effects of various living-low and training-high modes with distinct training prescriptions on sea-level performance: a network meta-analysis]”. Your valuable comments have provided a clear direction towards improving my research work.

Each of your suggestions has been taken into consideration and applied to revise the manuscript. Your insights triggered a rethinking process and the manuscript has undergone substantial revision. The content has been restructured and details have been added as necessary to address the concerns you’ve raised. The methodological and conceptual changes you suggested have significantly enhanced the clarity and quality of our research.

Your expertise and unequivocal clarity are commendable, and your inputs have enriched my manuscript. I believe it will be more engaging and impactful after having integrated your suggestions.

Thank you once again for your thoughtful and enlightening perspectives. It has been a valuable learning experience, and I look forward to more opportunities to learn from your expertise in the future.

Best regards,

Xinmiao Feng

Beijing sports university

**Response to Raphael Faiss, Academic Editor**

It is an honor for us to receive your letter. Your research in the field of sports has provided us with tremendous assistance. On behalf of my co-authors, we thank you for giving us a chance to revise and improve the quality of our article. Compared to our previous submission experiences, what impressed us is that the peer review process at PLOS ONE is so meticulous and rigorous. Every member of our team admires your work and we also appreciate your valuable suggestions. Finally, we would like to apologize to you as our team’s negligence has increased your workload.

(1) After initial review by myself and before submitting to the reviewers after your answers to their comment, i noticed a samll error in the "labelling" of the studies included (Table 2).

For several studies, you inversed first name and last name of the corresponding author.

Please correct for

Paul et a.

Margaux et al.

David et al.

Raphael et al.

Harvey et al.

Hannes et al.

Please note also that for the Faiss et al (2013) study with cyclists (labeled here as "Raphael et al.) , there were 20 subjects for N and 20 for H (not 25!) since there was a control group with no training of 10 subjects.

Response:

The aforementioned problem were due to the negligence of the team member responsible for the extraction of literature characteristics of RHS intervention. Firstly, we have rectified the error of "labelling" in Table 2. Secondly, we wholeheartedly thank you for pointing out the erroneous subjects number in this study conducted by Faiss et al. This error has been rectified in the abstract, results, Table 2, limitations, and supported information. Lastly, it is essential to acknowledge that the error in the inclusion subjects number could potentially impact the statistical results. Therefore, we have conducted a comprehensive review of all the data to address this issue. Fortunately, the modification made to the inclusion number in the study did not result in any significant deviation in the research findings (former statistical results before and modified results as follows). All of the above modification have been marked by red in the revised manuscript. These errors and defects are actually quite serious, but we are extremely grateful for the opportunity you have given us.

(1) Forest plot

Fig 1a. Former aerobic result

Fig 1b. modified aerobic result

Fig 2a. former anaerobic result

Fig 2b. modified anaerobic result

(2) League table

Table 1a. Former aerobic result

| ***l-IHT*** | . | . | . | . | . | . | **0.78  (0.52;1.05)** |
| --- | --- | --- | --- | --- | --- | --- | --- |
| **0.49  (0.16;0.82)** | ***RSH*** | . | . | . | . | -0.03  (-1.25;1.19) | **0.30  (0.10;0.50)** |
| **0.55  (0.09;1.00)** | 0.06  (-0.36;0.4) | ***s-IHT*** | . | . | 0.18  (-0.49;0.8) | . | 0.20  (-0.18;0.58) |
| **0.60  (0.18;1.01)** | 0.11  (-0.27;0.4) | 0.05  (-0.44;0.54) | ***C+I*** | . | . | . | 0.18  (-0.13;0.50) |
| **0.64  (0.27;1.01)** | 0.15  (-0.18;0.4) | 0.09  (-0.36;0.54) | 0.04  (-0.37;0.4) | ***IHE*** | . | . | 0.15  (-0.11;0.40) |
| **0.69  (0.20;1.17)** | 0.20  (-0.26;0.6) | 0.14  (-0.35;0.62) | 0.09  (-0.43;0.6) | 0.05  (-0.43;0.53) | ***CHT*** | . | 0.07  (-0.36;0.49) |
| **0.76  (0.22;1.30)** | 0.27  (-0.23;0.7) | 0.21  (-0.39;0.81) | 0.16  (-0.41;0.7) | 0.13  (-0.41;0.66) | 0.08  (-0.55;0.7) | ***ISH*** | -0.03  (-0.54;0.48) |
| **0.78  (0.52;1.04)** | **0.29  (0.09;0.49)** | 0.24  (-0.14;0.61) | 0.18  (-0.13;0.5) | 0.15 (-0.11;0.40) | 0.10  (-0.31;0.5) | 0.02 (-0.45;0.49) | ***CON*** |

Table 1b. modified aerobic result

| ***l-IHT*** | . | . | . | . | . | . | **0.78  (0.52;1.05)** |
| --- | --- | --- | --- | --- | --- | --- | --- |
| **0.49  (0.15;0.82)** | ***RSH*** | . | . | . | . | -0.03  (-1.25;1.19) | **0.31  (0.10;0.51)** |
| **0.55  (0.09;1.00)** | 0.06  (-0.36;0.48) | ***s-IHT*** | . | . | 0.18  (-0.49;0.85) | . | 0.20  (-0.18;0.58) |
| **0.60  (0.19;1.01)** | 0.11  (-0.26;0.49) | 0.05  (-0.44;0.54) | ***C+I*** | . | . | . | 0.18  (-0.13;0.50) |
| **0.64  (0.27;1.01)** | 0.15  (-0.18;0.48) | 0.09  (-0.36;0.54) | 0.04  (-0.37;0.4) | ***IHE*** | . | . | 0.15  (-0.11;0.40) |
| **0.69  (0.20;1.17)** | 0.20  (-0.25;0.65) | 0.14  (-0.35;0.62) | 0.09  (-0.43;0.6) | 0.05  (-0.43;0.53) | ***CHT*** | . | 0.07  (-0.36;0.49) |
| **0.76  (0.22;1.30)** | 0.28  (-0.23;0.78) | 0.21  (-0.39;0.81) | 0.16  (-0.41;0.73) | 0.12  (-0.41;0.66) | 0.08  (-0.55;0.7) | ***ISH*** | -0.03  (-0.54;0.48) |
| **0.78  (0.52;1.05)** | **0.30  (0.10;0.50)** | 0.24  (-0.14;0.61) | 0.18  (-0.13;0.5) | 0.15 (-0.11;0.40) | 0.10  (-0.31;0.5) | 0.02 (-0.45;0.49) | ***CON*** |

Table 2a. Former aerobic result

| ***l-IHT*** | . | . | . | . | . | **0.97  (0.12;1.82)** |
| --- | --- | --- | --- | --- | --- | --- |
| 0.56  (-0.36;1.49) | ***ISH*** | . | 0.32  (-0.97;1.61) | . | . | **0.38  (0.01;0.76)** |
| 0.65  (-0.24;1.54) | 0.09  (-0.36;0.54) | ***s-IHT*** | . | . | . | **0.32  (0.05;0.59)** |
| 0.65  (-0.23;1.53) | 0.09  (-0.32;0.50) | 0.00  (-0.35;0.35) | ***RSH*** | . | . | **0.32  (0.09;0.55)** |
| 0.87  (-0.09;1.82) | 0.30  (-0.26;0.87) | 0.21  (-0.30;0.73) | 0.21  (-0.28;0.70) | ***IHE*** | . | 0.10  (-0.33;0.54) |
| **2.13  (0.72;3.54)** | **1.57  (0.38;2.75)** | **1.48  (0.32;2.64)** | **1.48  (0.33;2.63)** | **1.26  (0.05;2.47)** | ***C+I*** | -1.16  (-2.29; -0.03) |
| **0.97  (0.12;1.82)** | **0.40  (0.04;0.76)** | **0.32  (0.05;0.59)** | **0.31  (0.09;0.54)** | 0.10  (-0.33;0.54) | -1.16 (-2.29; -0.03) | ***CON*** |

Table 2b. Former anaerobic result

| ***l-IHT*** | . | . | . | . | . | **0.97  (0.12;1.81)** |
| --- | --- | --- | --- | --- | --- | --- |
| 0.56  (-0.36;1.48) | ***ISH*** | . | 0.32  (-0.97;1.61) | . | . | **0.38  (0.01;0.76)** |
| 0.65  (-0.24;1.54) | 0.09  (-0.36;0.54) | ***s-IHT*** | . | . | . | **0.32  (0.05;0.59)** |
| 0.64  (-0.23;1.52) | 0.08  (-0.33;0.50) | -0.01  (-0.36;0.35) | ***RSH*** | . | . | **0.32  (0.10;0.56)** |
| 0.87  (-0.09;1.82) | 0.30  (-0.26;0.87) | 0.21  (-0.30;0.73) | 0.22  (-0.27;0.71) | ***IHE*** | . | 0.10  (-0.33;0.53) |
| **2.13  (0.72;3.54)** | **1.57  (0.38;2.75)** | **1.48  (0.32;2.64)** | **1.49  (0.37;2.63)** | **1.26  (0.06;2.47)** | ***C+I*** | -1.16  (-2.29;-0.04) |
| **0.97  (0.12;1.81)** | **0.40  (0.04;0.76)** | **0.32  (0.05;0.59)** | **0.32  (0.10;0.55)** | 0.10  (-0.33;0.53) | -1.16 (-2.29;-0.04) | ***CON*** |

**Response to Reviewer 1: Thank you for your review of our paper. We have answered each of your points below.**

Reviewer #1:

The manuscript is very interesting and well written. All comments and suggestions I provide below have the only purpose to increase the quality of the present work. I hope you find my suggestions useful.

Response: The authors would like to thank for reviewers’ extensive corrections and comments for the original manuscript. We are pleased to follow the reviewers’ suggestions and criticisms. The modified parts have been marked by red in the revised manuscript. In addition, the concerns raised by the reviewers are addressed point-by-point below.

Introduction

(1) Line 43-44: “most studies have focused …………..rest periods”. Authors cited only one reference. Please give more references.

Response:

We appreciate it very much for this good suggestion, and we have added the corresponding references [11, 12] in line 48 on page 3 of the manuscript, and already marked in red font.

(2) Line 56-58: I suggest authors to cite some of these studies.

Response:

We appreciate it very much for this good suggestion, and we have added the corresponding references [22, 23] in line 61 on page 3 of the manuscript, and already marked in red font.

(3) Line 68: Please check the spelling of “hypoxic”

Response:

We are very sorry for our incorrect writing and it is rectified at Line 70 on page of 4, and already marked in red font.

(4) Line 74: Change “Training of varying intensities and durations” to “training at different duration and intensities”.

Response:

We are very sorry for our incorrect writing and it is rectified at Line 76 on page of 4, and already marked in red font.

(5) Line 89: Please add “i.e., aerobic and anaerobic”.

Response:

We appreciate it very much for this good suggestion, and it is rectified at Line 86-87 on page of 4, and already marked in red font.

Methods

(6) Line 92: Change “review” to “meta-analysis” since it is a network meta-analysis.

Response:

We appreciate it very much for this good suggestion, and it is rectified at Line 90 on page of 5, and already marked in red font.

(7) Line 93-94: Change “This study has been registered” to “The study protocol has been registered”.

Response:

We appreciate it very much for this good suggestion, and it is rectified at Line 91-92 on page of 5, and already marked in red font.

(8) In your search terms you have not included "performance" which is in your main concern. How you justify this?

Response:

We appreciate it very much for this good suggestion, detailed search strategy is in Supporting information file 3, which contains search terms related to performance. We are sorry that we did not include this search term in the main text and it is rectified at Line 99 on page of 5, and already marked in red font.

(9) Line 102: Remove “Additionally”.

Response:

We appreciate it very much for this good suggestion, and we have removed this word.

(10) Line 107: Change “the past 6 months” to the last 6 months”.

Response:

We are very sorry for our incorrect writing and it is rectified at Line 105 on page of 5, and already marked in red font.

(11) Line 124-126: Please add “i.e., cold”.

Response:

We appreciate it very much for this good suggestion and it is rectified at Line 132 on page of 6, and already marked in red font.

(12) Line 126: Please add “i.e., nitrate, caffeine”.

Response:

We appreciate it very much for this good suggestion and it is rectified at Line 133 on page of 7, and already marked in red font.

(13) Line164-166: I suggest authors to add the reference about the tau squared (τ2).

Response:

We appreciate it very much for this good suggestion and we have added the corresponding references [44, 45] in line 177 on page 8 of the manuscript, and already marked in red font.

(14) Line 456-457: Please clarify this sentence “Neither passive hypoxic exposure (IHE) nor moderate-intensity CHT was proven effective”. The abbreviation IHE was defined previously as intermittent hypoxic exposure. If author use “IHE” as an example please add e.g., before.

Response:

We appreciate it very much for this good suggestion and it is rectified at Line 486 to 487 on page of 40, and already marked in red font.

Tables

(15) Table 1: Please add PEDro score of the included studies.

Response:

We appreciate it very much for this good suggestion and PEDro score has been added in Table 2.

(16) Table 1: Some abbreviations included in this table are not defined at the foot of the table (e.g., N, H, UCT). Please revise this.

Response:

We are very sorry for our negligence. The definition of these abbreviations at the foot of the table, and already marked in red font.

(17) Table 1: Author used the abbreviation is “C+T”, should this be “C+I”?

Response:

We are very sorry for our incorrect writing and it is rectified.

**Response to Reviewer 2: Thank you for your review of our paper. We have answered each of your points below.**

Reviewer #2

Thank you for providing me with the opportunity to review this study. This meta-analysis, which evaluates the effects of Living low training high across different exercise durations, presents very intriguing findings. Please address the following comments.

Response: The authors would like to thank for reviewers’ extensive corrections and comments for the original manuscript. We are pleased to follow the reviewers’ suggestions and criticisms. The modified parts have been marked by red in the revised manuscript. In addition, the concerns raised by the reviewers are addressed point-by-point below.

Major Comments:

(1) The manuscript is dense and represents a robust network meta-analysis; however, please reduce redundancy and ensure that essential information is included in the main text. For instance, while the Introduction contains some details unrelated to the current study's methods, key methodological points are written in the Supplementary File. Also, organize the information from the Supplemental section coherently within the main text. Details on the Supplemental comments are noted in the minor comments section.

Response:

Thank you for your valuable suggestions and acknowledgement. We have added parts of the content in the attachment to the main text, such as the naming and definition of intervention measures. Thank you for this suggestion, this issue is indeed what we have been struggling with when submitting the article. Your advice has given us great support and confidence.

As for the problem of lengthy expression, we have made maximum revisions to the new manuscript.

(2) Abbreviations like RSH and IHT seem to be cited from previous studies; presenting them as a list makes it difficult to understand due to inconsistent use of initialisms and terminology, some including 'Hypoxia' and others 'Training'. I strongly recommend revising for clarity. Additionally, as these definitions are crucial to the paper, include them in the main text rather than in the supplemental material.

Response:

We wish to thank you and the reviewers for your insightful comments. These have greatly helped us to improve the quality of our manuscript. Regarding the abbreviation issue of certain hypoxia variants, your mentioned viewpoint has gained significant recognition from our team.

we have revised the abbreviation of “intermittent sprint in hypoxia” from “SIH” to “ISH”, but for other abbreviations used in this manuscript are consistent with the authoritative reviews or panorama studies published in recent years [1-4]. Additionally, our team has previously conducted related research using this abbreviation paradigm [5]. Unfortunately, it is regrettable that we are unable to modify these abbreviations. However, if you could assist us by providing more convenient and comprehensive abbreviations for repeated hypoxic sprints and high-intensity intermittent hypoxic training, we would greatly appreciate it and immediately apply them. Given our current academic background and capabilities, it is challenging for us to make such modifications. We apologize for any inconvenience caused.

In addition, our team strongly agrees with the suggestion of transferring the definition of intervention measures to the main text. This issue has been bothering us since before submission, and your viewpoint has given us great confidence and assistance. The definition table 1 has been added at line 109 on page of 5 and line 135 on page 7.

(3) There is only one study on the improvement of anaerobic performance by L-IHT (Table 1), so it is questionable to treat this as a main finding.

Response:

We wish to thank you and the reviewers for your insightful comments. We have already added related content to the results, discussion and limitations sections, the content shows the lack of robustness of this situation and its corresponding results, at Line 274 to 275 on page of 29, Line 395 to 399 on page of 35-36, Line 476 to 481 on page of 39 and already marked in red font.

(4) Most studies on L-IHT involve endurance athletes. Please mention how the type of training modality may influence the characteristics of the athlete in the discussion, or at the very least, as a limitation.

Response:

We appreciate it very much for this good suggestion. In this study, although the majority of subjects included were endurance athletes, some were team-sports. In addition, characteristics such as the athletic level and gender of these athletes were not uniform. In theory, we could address this issue through subgroup analysis, but this would significantly reduce the number of related studies and severely undermine the credibility of the results and the network connectivity of meta-analysis, as well as easily lead to misinterpretation. Therefore, following your second suggestion, we have added this perspective to the limitations section at Line 470 to 475 on page of 39, and already marked in red font.

(5) Please add a discussion on the mechanism by which RSH may improve aerobic performance.

Response:

We appreciate it very much for this good suggestion. This is a very good suggestion, pointing out a key oversight in this section, which makes the meaning of the section unclear. Past research suggests that oxidative tendencies of fast muscle fibers are believed to be associated not only with anaerobic performance improvements, but also aerobic performance improvements (relevant arguments and citations are in the manuscript). But given the methodological characteristics and results of the network meta-analysis (RSH’s effect is not optimal), we did not discuss it too much in the text. However, in order to increase the rigor of the study and prevent incorrect interpretations by the reader, we have had a new, more detailed discussion about the relevant mechanisms of RSH, at Line 445 to 451 on page of 38, and already marked in red font.

Minor Comments:

(6) In the Abstract, please describe the duration of exercise for each exercise modality.

Response:

We appreciate it very much for this good suggestion, and we have added it in the Abstract, and already marked in red font.

(7) Line 68, remove the unnecessary spaces.

Response:

We appreciate it very much for this good suggestion, and we have rectified it at Line 70 on page of

4, and already marked in red font.

(8) Lines 80-83, since the study focuses on performance, there is no need to discuss health.

Response:

We appreciate it very much for this good suggestion, and we have rectified it.

(9) Line 163, please format “squared” as a superscript.

Response:

We appreciate it very much for this good suggestion, and we have rectified it and similar place at Line 176 on page of 9, and already marked in red font.

(10) Specify which performance indicators were used in the main text and appropriately cite Table S6.

Response:

Thanks for the constructive advice, and we have add it at Line 115 to 127 on page of 6, and already marked in red font.

(11) Since Supplement S5 is short, include it in the main text.

Response:

Thanks for the constructive advice, and we have rectified them at Line 147 to 150 on page of 8, and already marked in red font.

(12) In Table 1, use 'sports' instead of 'subject', and ensure uniformity by using the names of the sports (i.e., swimming) instead of participant types (i.e., swimmers).

Response:

Thanks for the constructive advice, and we have rectified them and similar place at Table 2, and already marked in red font.

(13) In Table 1, the Hypoxic protocol is also mentioned in S8. Please delete S8.

Response:

Thanks for the constructive advice, the difference between S8 and Table 1 is that the former has research citations. To limit the volume of citations in the manuscript, we put them in S8.

(14) In Table 1, it is Kasai et al., not Nobukazu et al. (Supplemental reference 33), and the participants were lacrosse players, not cyclists.

Thanks for the constructive advice, and we have rectified them at Table 2, and already marked in red font.

**Reference**

1.	Millet GP, Roels B, Schmitt L, Woorons X, Richalet JP. Combining hypoxic methods for peak performance. Sports Med. 2010;40(1):1-25. Epub 2009/12/22. doi: 10.2165/11317920-000000000-00000. PubMed PMID: 20020784.

2.	Levine BD. Intermittent hypoxic training: fact and fancy. High Alt Med Biol. 2002;3(2):177-93. Epub 2002/08/07. doi: 10.1089/15270290260131911. PubMed PMID: 12162862.

3.	Faiss R, Girard O, Millet GP. Advancing hypoxic training in team sports: from intermittent hypoxic training to repeated sprint training in hypoxia. Br J Sports Med. 2013;47 Suppl 1(Suppl 1):i45-50. Epub 2013/12/07. doi: 10.1136/bjsports-2013-092741. PubMed PMID: 24282207; PubMed Central PMCID: PMCPMC3903143.

4.	McLean BD, Gore CJ, Kemp J. Application of 'live low-train high' for enhancing normoxic exercise performance in team sport athletes. Sports Med. 2014;44(9):1275-87. Epub 2014/05/23. doi: 10.1007/s40279-014-0204-8. PubMed PMID: 24849544.

5.	Feng X, Zhao L, Chen Y, Wang Z, Lu H, Wang C. Optimal type and dose of hypoxic training for improving maximal aerobic capacity in athletes: a systematic review and Bayesian model-based network meta-analysis. Front Physiol. 2023;14:1223037. Epub 2023/09/25. doi: 10.3389/fphys.2023.1223037. PubMed PMID: 37745240; PubMed Central PMCID: PMCPMC10513096.

**Response to Reviewer 3: Thank you for your review of our paper. We have answered each of your points below.**

Reviewer #3

First of all, the reviewer would like to thank the authors for their work and efforts in trying to improve sports science knowledge. The article is an interesting approach to the ffects of various living-low and training-high modes with distinct training prescriptions on sea-level performance. The study is well designed and well-written, with a great introduction proposing the usefulness of the topic and a clear outline of the research question. I suggest that the this article can be accepted without any revisions.

Response: Thank you for your recognition of our manuscript and we will continue to work hard on research in the field of sports.
