# Supplementary material for: Effects of various living-low and training-high modes with distinct training prescriptions on sea-level performance: A network meta-analysis
Source: PLoS One. 2024 Apr 18;19(4):e0297007. doi: 10.1371/journal.pone.0297007 (PMC11025749; doi:10.1371/journal.pone.0297007)
Supplement: Attachment — Submitted filename: Response to Reviewers.docx [file pone.0297007.s013.docx]

**Response to Reviewers**

**Response to Reviewer 1: Authors have adequately addressed my comments raised in a previous round of review. I feel that this manuscript is now acceptable for publication.**

Response: thanks for your careful checks and supports.

**Response to Reviewer 2: The manuscript has shown improvement, and I believe it meets the standards for publication in PLOSONE with a few minor modifications.**

Reviewer #2

We feel great thanks for your professional review work on our article. As you are concerned, there are several problems that need to be addressed. According to your nice suggestions, we have made extensive corrections to our previous draft, the detailed corrections are listed below.

(1) Page 3, Lines 56-57: The phrase 'repeated sprint training in hypoxia' is mentioned.

Response: thanks for your careful checks. We have made a correction to the phrase, at at Line 56 to 57 on page of 3 and already marked in red font.

(2) Page 3, Line 58: The term 'interval sprint training in hypoxia' is used.

Response: thanks for your careful checks. We have made a correction to the phrase, at at Line 58 on page of 3 and already marked in red font.

(3) Page 6, Lines 115-127: There are several concerns in the revised section:

The term “al” is unclear. Could you please clarify what this refers to?

Please format the manuscript properly. Ensure there is a space between numbers and symbols.

The unit notation is ambiguous. The term 's/min' could be interpreted as seconds or minutes, but it currently appears as if it means 'second divided by minute'. Please clarify.

Response: thanks for your careful checks. We are sorry for our carelessness. Based on your comments, we have made the corrections, that “al” is wrong spell, formt and the expression of “s/min” has been modified, at at Line 116 to 128 on page of 6 and already marked in red font.
